# Cost-Effective Fabrication of Laser-Induced Graphene Electrochemical Cell for NADH Detection

**DOI:** 10.1021/acsomega.5c04282

**Published:** 2025-10-09

**Authors:** Ketley Caroline Rocha Pereira, Elsa Maria Materón, Matheus Santos Dias, Tatiana Parra Vello, Deissy Feria Garnica, Gustavo Miguel Sousa, Camila Marchetti Maroneze, Cecilia de Carvalho Castro Silva

**Affiliations:** † School of Engineering, 42524 Mackenzie Presbyterian University, São Paulo, 01302-907 São Paulo, Brazil; ‡ MackGraphe−Mackenzie Institute for Research in Graphene and Nanotechnologies, Mackenzie Presbyterian Institute, São Paulo, 01302-907 São Paulo, Brazil

## Abstract

The unique properties and versatile applications of laser-induced graphene (LIG) have garnered significant interest for electrochemical sensing technologies. In this study, we report the fabrication and application of an in-house produced LIG/polyimide (PI) composite, generated via 450 nm laser irradiation, for the amperometric detection of Nicotinamide Adenine Dinucleotide (NADH), a critical biomarker associated with several neurodegenerative human diseases. The LIG structure was confirmed by Raman spectroscopy, X-ray photoelectron spectroscopy (XPS), and sheet resistance measurements, with an average sheet resistance (*R*_s_) of 24.38 ± 2.19 Ω/□, indicating excellent electrical conductivity. XPS analysis revealed the presence of C=O bonds (288.9 eV), formed under oxidizing conditions during LIG fabrication, which may contribute to enhanced electrocatalytic activity by facilitating NADH oxidation through redox mediation. Using a printed Ag/AgCl pseudoreference electrode and an applied potential of only 50 mV vs Ag/AgCl, NADH was detected within a concentration range of 5 μmol L^–1^ to 10 mmol L^–1^. The sensor exhibited a limit of detection (LOD) of 2.72 μmol L^–1^ and a limit of quantification (LOQ) of 9.07 μmol L^–1^, with a linear response up to 1 mmol L^–1^. The repeatability of the electrochemical oxidation of NADH resulted in a relative standard deviation (RSD) of 2.76%, while the reproducibility, evaluated as intra- and interbatch variability, yielded RSD values of 5.78 and 8.22%, respectively. Furthermore, the total material cost for each electrochemical cell was estimated at only U$ 0.10, highlighting the method’s potential for low-cost and environmentally friendly biosensor development. The fabricated LIG platform offers a promising route for sensitive, scalable, and sustainable detection of NADH and potentially other clinically relevant analytes.

## Graphical abstract

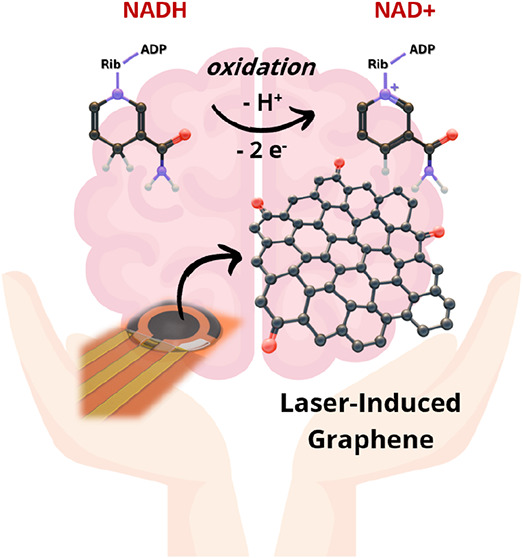

## 1 Introduction

Current diagnostic techniques often require specialized personnel, intricate laboratory setups, and expensive reagents and solvents.^1,2^ Undoubtedly, the imperative for point-of-care solutions cannot be overstated, owing to their remarkable attributes of swift response and high sensitivity. In light of this demand, wearable and flexible disposable electrochemical sensors and biosensors have emerged as promising alternatives, offering affordability, potential for miniaturization, low cost, and compatibility with eco-friendly methodologies.^3–5^

Graphene derivative materials are often used as an ideal electrode material for the development of electrochemical sensors^6^ due to their excellent electrical (carrier mobility ∼250,000 cm^2^ V^–1^ s^–1^) and mechanical (Young’s modulus: 1 × 10^12^ Pa) properties, large surface area (2630 m^2^ g^–1^), chemical stability and biocompatibility.^7–10^ Conventionally, graphene derivative materials with some degree of structural defects, such as dangling bonds, vacancies, exposed edges, or even a limited presence of oxygen functional groups, demonstrate enhanced electrochemical activity (fast heterogeneous electron transfer (HET)) in electrochemical sensors and biosensors.^10–13^ In this context, reduced graphene oxide (rGO) has been deeply explored in the development of electrochemical sensors^14–16^ and biosensors.^17–19^ Despite notable progress in the large-scale solution process of graphene oxide (GO), the utilization of rGO often involves the complex synthesis of GO. This process traditionally requires strong chemical reagents for oxidizing graphite to GO, as the Hummers’ method,^20^ leading to the generation of a substantial volume of acidic residues that demand considerable energy and operational costs for recovery and treatment.^21^ Moreover, releasing toxic fumes such as N_2_O_4_ and NO_2_ into the atmosphere, along with ion leaching into water (Na^+^ and NO_3_^–^), raises environmental concerns.^22^ Besides the manufacturing process of GO dispersion, it is imperative to fabricate a film with precise control over the thickness and orientation of the flakes on the target substrate before the reduction process.^21^ Various surface-based coating techniques, such as dip coating, spin coating, drop-casting, Langmuir–Blodgett method, and electrophoretic deposition, have been investigated for this purpose.^23,24^ However, scaling up these methods remains challenging. Finally, to obtain rGO, it is necessary to undergo the reduction process of GO, which can be achieved through various approaches, but conventionally through thermal treatment at high temperatures and/or through the use of hazardous chemical reduction agents (e.g., hydrazine, hydroiodic acid, and sodium borohydride),^23^ representing high energy consumption and environmental impact.

Despite intensive research efforts, achieving chemical-free, low-temperature, and cost-effective processing of high-quality graphene derivative materials remains a significant challenge. In this way, researchers investigate alternative synthesis methods for graphene-based conductive materials. These methods aim to minimize ecological impact and waste generation and facilitate mass production while enhancing technological accessibility. In this scenario, laser-assisted processing methods have emerged as a powerful technique across diverse applications, spanning from materials processing to device fabrication. Particularly, laser-direct writing (LDW) distinguishes itself as a mask-less, catalyst-free, harmless, and noncontact approach, facilitating adaptable, swift, direct, and effective fabrication of intricate structures at macro-, micro-, and nanoscales utilizing laser technology.^25^ Significantly, LDW has been used in numerous investigations to transform GO into graphene derivative^26^ aiming at the development of different electrochemical and electrical devices, from sensing to energy storage.^17–19,26,27^ For instance, in 2014, James Tour’s research team at Rice University introduced a technique for producing three-dimensional (3D) porous graphene, denominated laser-induced graphene (LIG), by directly converting polymeric substrates into graphene through LDW.^28^ LIG production stands out as a one-step process that eliminates the need for high-temperature conditions, solvents, or subsequent treatments.^29,30^ It offers high precision, reproducibility, scalability (from nanomicro to macro dimensions), industrial feasibility, affordability, rapidity, large surface areas, eco-friendliness, ease of pattern creation, and excellent thermal and electrical conductivity.^30^ Furthermore, this methodology allows for the incorporation of metallic precursors to facilitate the simultaneous formation of nanoparticles and graphene-based materials, reducing the number of modification steps in the devices and enabling fast production.^31^

Laser-scribed polyimide materials have been widely explored, but it has been demonstrated that most of other polymer source materials undergo ablation when irradiated with a laser at room temperature.^32,33^ This opens up the possibility of utilizing nonpolymers, paper, metal/plastic composites, biodegradable materials, naturally occurring substances, and even foods as platforms for generating LIG.^34,35^ The laser transforms a suitable polymeric substrate into LIG through a photothermal pyrolysis process. Various laser types, including excimer (248 nm), CO_2_ (10.6 μm), a low-energy laser diode (780 nm), near-IR (NIR) lasers (1070 nm), and neodymium-doped yttrium aluminum garnet laser (1064 nm), have been employed in LIG methodology.^30^ However, the high intrinsic cost of some lasers severely restricts their widespread use in mass manufacturing LIG-based applications, limiting them primarily to laboratory settings. Numerous studies documented in the literature have employed LIG for detecting a range of analytes, including uric acid,^36^ *Salmonella enterica* in chicken broth,^37^ sulfanilamide,^38^ nitrogen,^39^ paraquat,^40^ and atrazine pesticides,^41^ among others.

In this study, we present a cost-effective, straightforward, one-step fabrication method for producing flexible electrochemical sensors utilizing a simple, low-cost laser engraver machine (450 nm), exploring a low power consumption of energy (295 mW) and polyimide (PI) as a polymer source, in a complete integrated electrochemical cell. As a proof of concept, we employed the versatile electrochemical sensor based on LIG to detect Nicotinamide Adenine Dinucleotide (NADH), a crucial biomarker involved in metabolic redox reactions within living cells.^42^ Ultimately, this methodology enables the fabrication of conductive materials for many applications.

## 2 Experimental Section

### 2.1 Laser-Induced Graphene-Based Sensor Fabrication

First, the LIG was obtained on PI with 125 μm-thick substrates (FlexFilm, Brazil), using a pulsed laser (laser wavelength of 450 nm, frequency of 20 kHz), under conditions of 295 mW laser power, engraving speed of 8 mm/s, and one processing step, in an air atmosphere. A control experiment was conducted to prepare the surface of LIG electrodes under varying laser conditions, specifically using a laser power of 82.5 mW, an engraving speed of 17 mm/s, and two processing passes, all performed in ambient air. The laser power was measured using a Newport 1935-C power meter (minimum resolution: 1 pW), coupled to a Thorlabs 1W optical fiber (Model 5401C, S/N 00114 WARL, spectral range: 0.2–10.6 μm).

The electrochemical LIG-based sensors were manufactured following the procedure illustrated in Scheme 1. The electrochemical cell was composed of three electrodes. The working (geometric area = 0.096 cm^2^) and counter electrodes were made by LIG. The metal contact lines and silver reference electrode were obtained using silver ink (SPI-ink), deposited using a regular brush over the polyimide, and a shadow mask delimited the LIG area. A curing process was performed in a hot plate (Tecnal) at 120 °C for 20 min to remove the excess solvent from the silver ink. To delimit the active area of the electrodes and avoid interference response from exposed silver contact lines, a layer of nail polish was applied between the electric contacts and electrodes (the work, counter, and pseudo reference, respectively), followed by passivation with Kapton tape. Finally, the Ag/AgCl pseudo reference electrode was manufactured based on the work reported by Silva et al.^43^ A commercial bleach solution (2.5% (v/v) sodium hypochlorite) was dripped over the reference silver electrode area for 10 min, followed by Milli-Q water rinsing and dried by a Nitrogen stream.

**Scheme 1:**
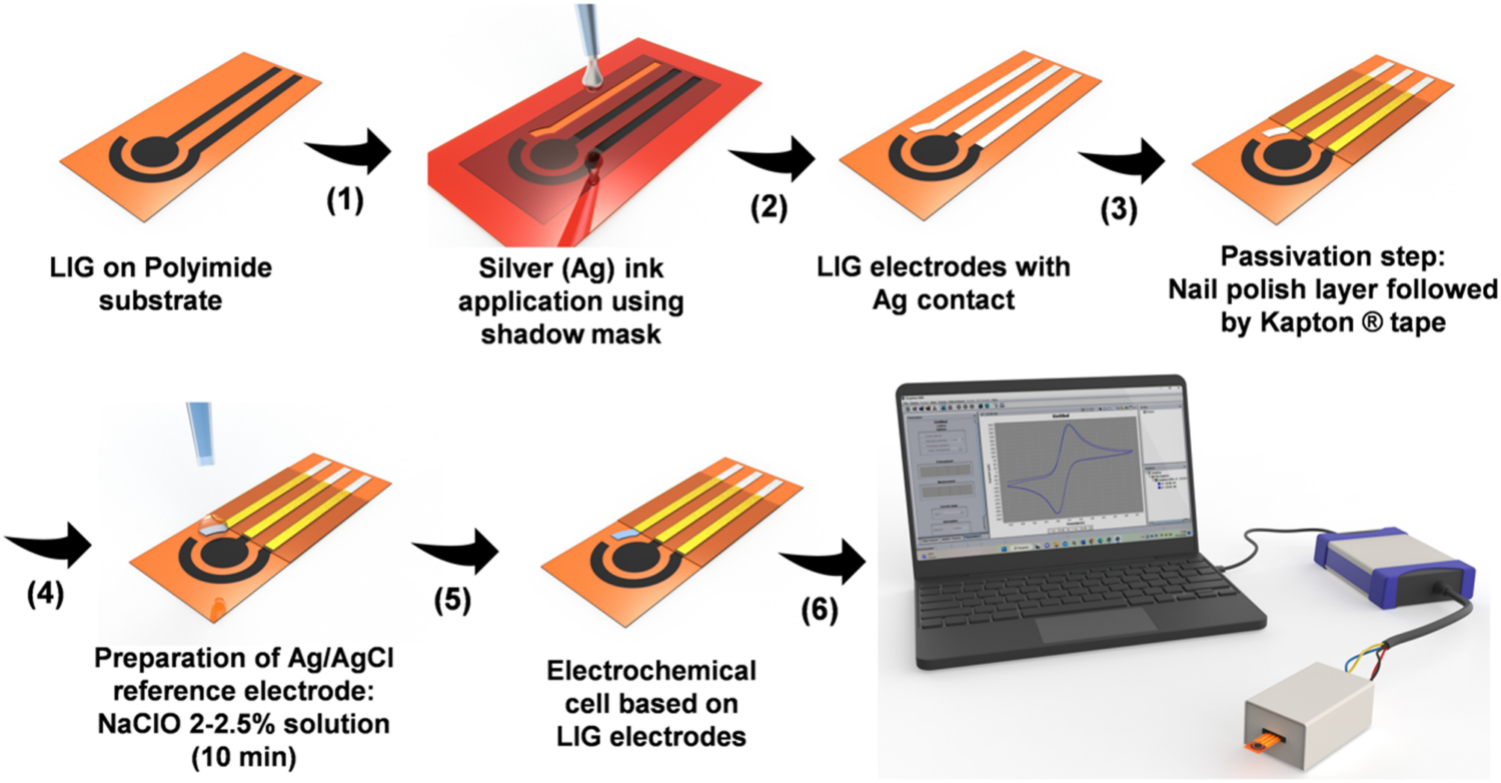
Schematic Representation of the Laser-Induced Graphene Sensors Fabrication Process

### 2.2 Laser-Induced Graphene Characterization

LIG’s structure was characterized through Raman spectroscopy coupled to a confocal optical microscope, Witec α 300R. The measurement was performed using a 532 nm laser source with a power of 1.5 mW and a microscope objective of 50× magnification. The spectra were acquired with 10 s of integration time and an accumulation of 10 spectra.

X-ray photoelectron spectroscopy (XPS) experiments (survey and high-resolution C 1s spectra), were performed with a Thermo Scientific K-Alpha spectrometer. Two different areas of the LIG’s sample were analyzed, and all spectra were taken using an Al Kα microfocused monochromatized source with a resolution of 0.100 eV, pass energy of 50 eV, and a spot size of 300 μm. The peak fitting was performed using the Avantage software (version 6.6).

For the electrical characterization, circular samples (1 cm of diameter) were obtained, and the values of LIG sheet resistance (Ω/□) were analyzed by the Osilla Four-Point probe system, with a maximum current range applied of 100 μA. Six different samples of LIG were analyzed in two to four different areas of each sample. The morphology of the LIG was characterized by field-emission scanning electron microscopy (SEM) (Jeol, model JSM-7800).

The fabricated LIG sensors and Ag/AgCl pseudoreference electrodes were electrochemically characterized. First, the quality of the pseudoreference electrode was evaluated by chrono potentiometric analysis, using a potentiostat from Autolab (PGSTAT204) in a solution of KCl 3 mol L^–1^ and a standard external reference Ag/AgCl (3 mol L^–1^ KCl) electrode. The long-term stability of the Ag/AgCl pseudoreference electrodes was evaluated over a one-year period by analyzing 12 electrodes, with measurements performed on three different electrodes at each time point using the same experimental procedure. To preserve their integrity, the electrodes were stored in a desiccator under vacuum conditions (−400 mmHg) between measurements.

In addition, the electrochemical behavior of the LIG sensors was characterized by cyclic voltammetry employing Fe­(CN)_6_^2–/3–^ at 10 mmol L^–1^ in KCl 3 mol L^–1^ as a redox probe.

### 2.3 Electrochemical Detection of NADH

The fabricated laser-induced graphene-based sensors were tested for NADH detection as proof of concept. For this, cyclic voltammetry was performed on a blank sample, and a 1 mmol L^–1^ NADH solution was prepared in a 100 mmol L^–1^ sodium phosphate buffer (pH 7.4) at a scan rate of 10 mV/s. Chronoamperometry was employed to quantify NADH in 100 mmol L^–1^ sodium phosphate buffer (pH 7.4) and measured at 50.0 mV vs a printed Ag/AgCl pseudo reference electrode. The current signal for each concentration was recorded at 150 s. Additionally, results from cyclic voltammetry were compared with those obtained from a commercial screen-printed carbon electrode (SPCE), under the same experimental conditions described previously.

Selectivity studies were conducted by evaluating potential interfering compounds commonly present in whole blood, specifically glucose and urea, at fixed concentrations of 5 mM and 7 mM, respectively, values that correspond to their normal physiological levels.^44^ Chronoamperometric measurements were carried out in triplicate (*n* = 3) using a 0.1 M phosphate-buffered saline (PBS) solution at pH 7.4. A potential of +50 mV vs Ag/AgCl was applied to LIG-based electrodes for the electrochemical detection of 1 mM NADH under different conditions: in the presence of 5 mM glucose, 7 mM urea, and both interferents simultaneously.

## 3 Results and Discussion

Figure 1(a) presents a schematic representation of LIG nanomaterials synthesis on PI polymer substrate. The laser scribing process promotes the fabrication of porous carbon nanostructures by simultaneously breaking the carbon bonds in PI, thereby embedding and patterning them into LIG shaped electrode.^31^ The porous structure of LIG is confirmed by the SEM images in Figure 1(b i-ii), which reveal the material’s surface. The high-magnification image (Figure 1b-ii) illustrates the microporosity of the LIG material, exhibiting a random aspect due to the high local heating and thermal expansion induced by laser irradiation during the conversion of PI to LIG.^45^

**Figure 1:**
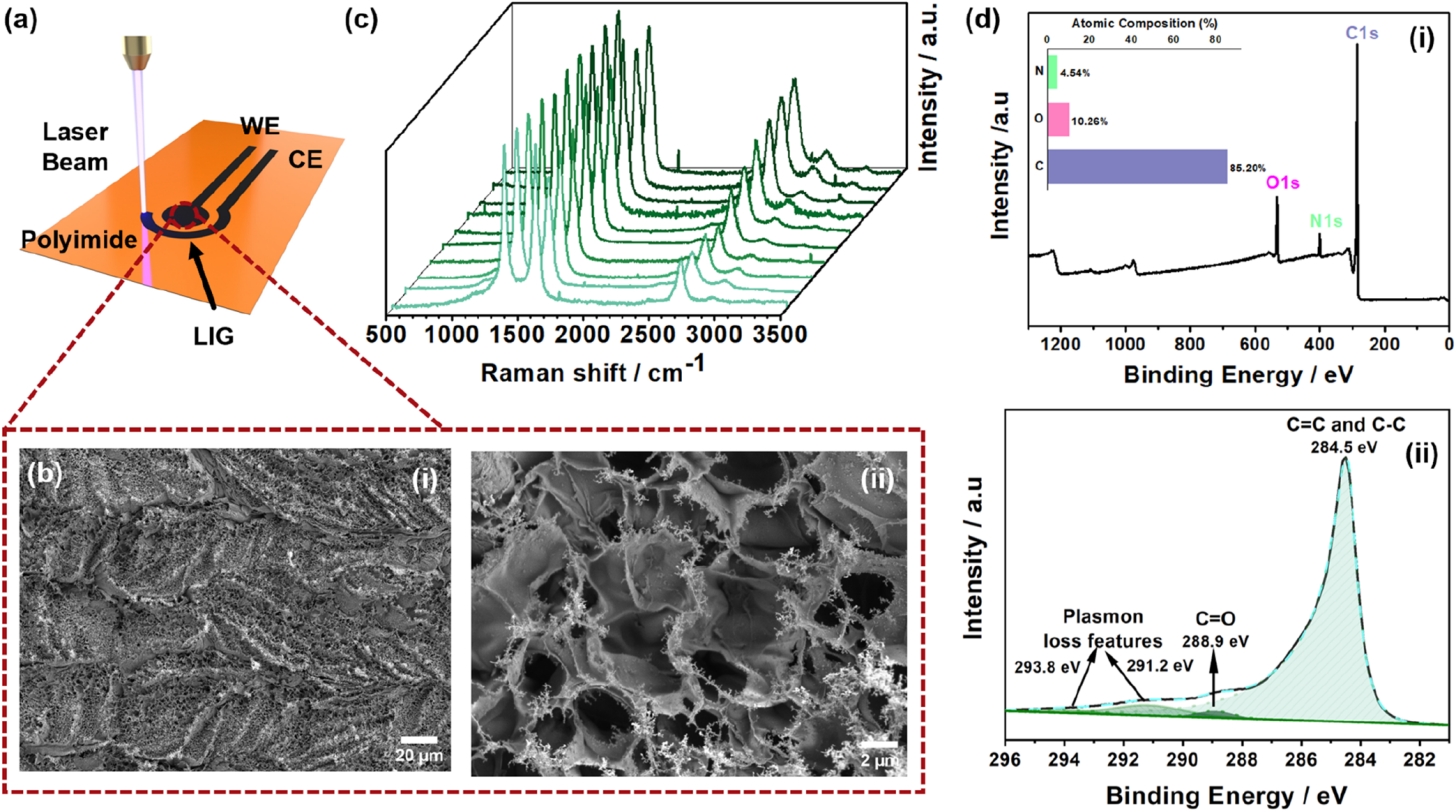
(a) Schematic representation of LIG sensor, working electrode (WE) and counter electrode (CE) obtained on Polyimide substrate by laser irradiation. (b) SEM images of LIG surface morphology in different magnifications (i and ii). (c) Representation of the obtained Raman spectra of LIG in different sample areas in multiple samples. (d) Representative XPS analysis of LIG samples: survey spectrum with elemental composition analysis (i) and high-resolution spectrum for C 1s (ii).

Figure 1(c) exemplifies the characteristic Raman spectra of the LIG obtained from different prepared samples. The graphene-derived materials exhibit three characteristic bands. The D band, around 1350 cm^–1^, suggests a structural disorder in the basal plane of the sample, indicating possible defects in the sheet plane or edge defects (dangling bonds) and residual oxygen functional groups. The G band, at 1580 cm^–1^, has its position and intensity directly related to the degree of graphitization in sp^2^ carbon atom structures, attributed to the stretching mode of C=C. Lastly, the two-dimensional (2D) band, near 2700 cm^–1^, suggests the level of organization in the bidimensional plane of the structure.^46^

The observed high intensity of the D band relative to the G band in the Raman spectra was confirmed by the high mean value of the intensity ratio of D and G bands (*I*_D_/*I*_G_) of 1.13 ± 0.15 (*n* = 10), indicating a high defect density in the formed graphitic structure, possibly due to the low power of the laser used in the air atmosphere, which may contribute to a lower level of graphitization degree and the presence of some oxygen functional groups.^35,47^ The low intensity of the 2D band is related to the structural disorder of the bidimensional plane of LIG due to the porous structure of this material.^31^ Despite these characteristics, slight deviations in the band intensities were observed in the Raman features, which may be related to the laser path. In some areas, there may exist an overlap between the LIG layers when the second laser scanner interacts with the borders of the LIG formed in the previous scan, altering some structural aspects of the LIG in these specific areas.^48^

XPS measurements were performed to evaluate the atomic composition of LIG electrodes. Figure 1(d - (i)) shows the survey XPS spectrum of the representative LIG sample confirming the dominant presence of carbon atoms (C 1s) at 285.07 eV and 85.21%, followed by oxygen (O 1s) at 532,78 eV and 10.26%, and nitrogen (N 1s) at 400.08 eV and 4.54%. Nitrogen atoms are related to some residues of C–N bonds from polyimide that were not fully converted during the laser irradiation to LIG.^49,50^ The C 1s high-resolution XPS spectra presented in Figure 1(d - (ii)) shown the four bonding modes and their approximate binding energies: C=C/C–C (284.5 eV), C=O (288.9 eV) and π–π shakeup satellite peaks characteristic of sp^2^-hybridized carbon (291.2 and 293.8 eV), which reveal the crystalline nature of carbon atoms on the LIG structure.^51–53^ The band observed at 288.9 eV confirms the presence of C=O bonds within the LIG structure, formed during the laser-induced graphene fabrication process in an air environment (an oxidizing atmosphere).^54^ This feature is particularly valuable for the electrochemical performance of LIG-based sensors, as carbonyl oxygen groups can act as redox mediators in the electrocatalytic oxidation of specific biomolecules, such as NADH. Carlson and Miller were among the first to demonstrate the mechanism of NADH oxidation mediated by quinones,^55^ and later, de Camargo et al. further explored the role of these functional groups in enhancing the electrocatalytic oxidation of NADH on electrochemically reduced graphene oxide-based electrodes.^56^

The electric aspects of formed LIG were investigated by a four-point probe system, which indicates an extremely important parameter for attesting the electric quality of materials applied to the development of electrochemical sensors. Regarding this, six different samples of LIG in PI substrates were evaluated, and different areas of the samples were measured, totalizing 20 sheet resistance measurements, enabling the sheet resistance (*R*_s_) mean value of 24.38 ± 2.19 Ω/□. This low value obtained characterizes the material as having excellent electrical conductivity in comparasion with other works reported in the literature that obtained LIG from PI.^57–62^ Despite the high density of structural defects revealed by Raman spectroscopy (indicated by the strong intensity of the D band), the LIG produced under these conditions exhibits good electrical percolation and excellent electrical conductivity. This is attributed to the use of a low-wavelength (450 nm), low-power (295 mW) laser, which enables the formation of C sp^2^ domains without reaching excessively high local temperatures. As a result, the surface roughness of the LIG is reduced, further enhancing electrical percolation.^63^

The fabricated LIG-based sensors were electrochemically characterized. Primarily, the quality of the pseudoreference electrode was evaluated through chronopotentiometric analysis, comparing the manufactured electrode with a commercial Ag/AgCl (3 mol L^–1^ KCl), as shown in Figure 2(a). The potential variation between the commercial electrode and the manufactured electrode obtained was under 1.5 (±0.5) mV in 800 s, demonstrating excellent stability and the effectiveness of the method in producing a pseudoreference electrode with performance comparable to that of a commercial one. The long-term stability of the fabricated Ag/AgCl pseudoreference electrodes was evaluated over a period of one year by analyzing 12 samples, as shown in Figure S1. After one year, the open-circuit potential (OCP) exhibited a slight variation, changing from 1.5 ± 0.5 to 2.2 ± 1.9 mV, indicating good stability and reproducibility of the pseudoreference electrodes under the tested storage conditions.

**Figure 2:**
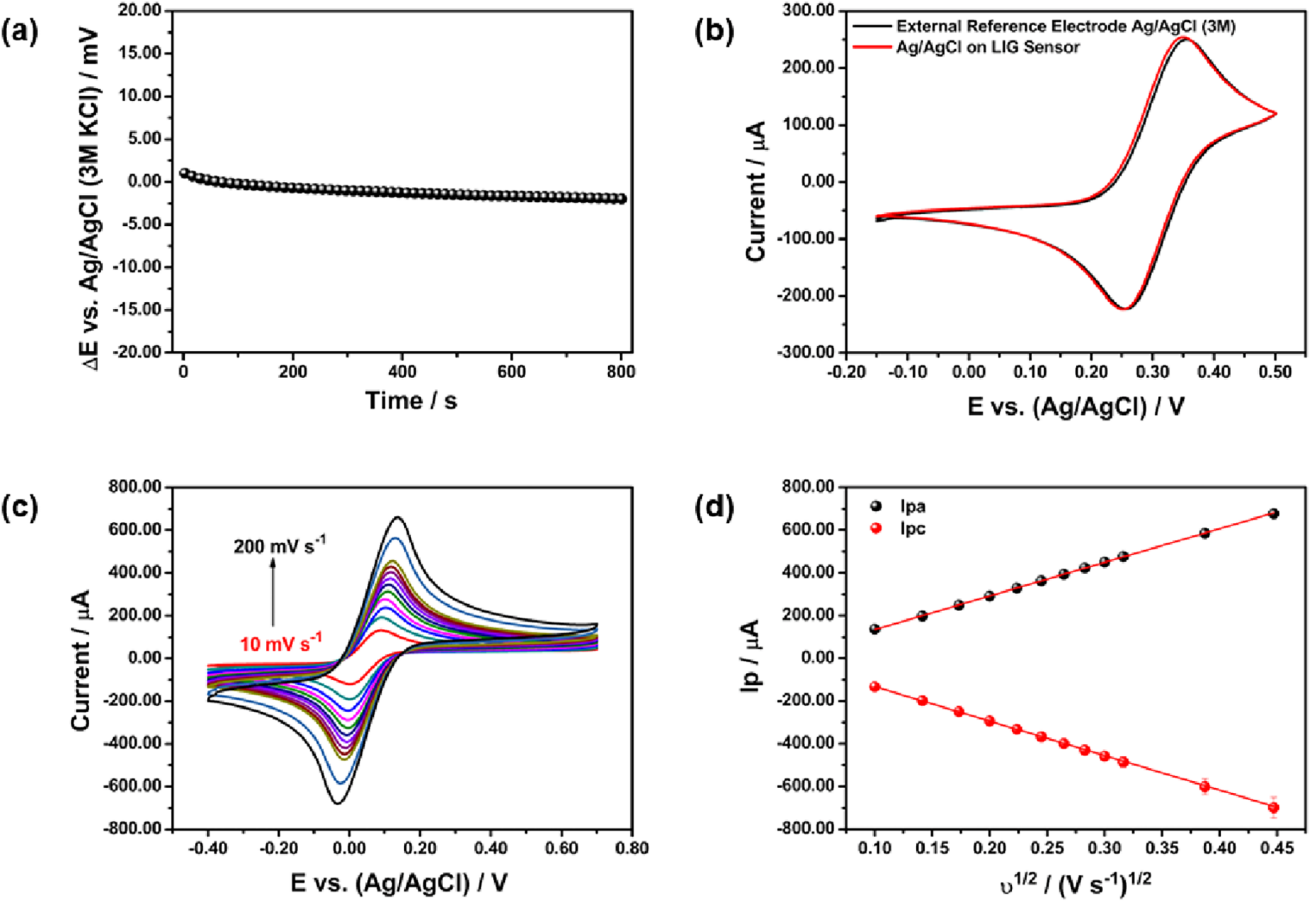
(a) Chronopotentiometric analysis of fabricated reference electrode vs commercial Ag/AgCl (3 mol L^–1^ KCl) reference electrode recorded in KCl (3 mol L^–1^). (b) Cyclic voltammetry of LIG sensor fabricated reference electrode vs commercial Ag/AgCl (3 mol L^–1^ KCl) reference electrode in 10 mmol L^–1^ Fe­[(CN)_6_]^3–/4–^ 3 mol L^–1^ KCl solution. Scan rate: 30 mV/s. (c) Cyclic voltammetry of LIG sensor in 10 mmol L^–1^ Fe­[(CN)_6_]^3–/4–^ 3 mol L^–1^ KCl solution (Scan rate: 10 to 200 mV/s). (d) Linear relationship of the anodic (Ipa) and cathodic (Ipc) peak currents as a function of the square root of the potential scan rate.

To better understand the electrochemical behavior of manufactured pseudoreference electrode, cyclic voltammetry experiments were performed in Ferri/Ferro redox couple (Fe­[(CN)_6_]^3–/4–^) 10 mmol L^–1^ in KCl (3 mol L^–1^) electrolyte (which is sensitive to electrode surface) compared to a commercial Ag/AgCl reference electrode. Figure 2(b) exhibits the voltammetric profile of the LIG sensor with a manufactured pseudoreference electrode (in red) in comparison to the commercial reference electrode (in black).

As presented, the voltammetric profile overlap demonstrates that the manufactured pseudoreference electrode of the sensor has a very similar behavior compared to a commercial sensor.

For a deeper evaluation of the electrochemical behavior of the electrode surface, experiments using the Ferri/Ferro redox couple with different scan rates were done with values between 10 and 200 mV/s, as demonstrated in Figure 2(c). The scan profile indicates an increase of the oxidation and reduction peak currents along with the increase of the scan rate.

The obtained values of oxidation (Ipa) and reduction (Ipc) peak current were plotted as a function of the scan rate square root (v^1/2^), aiming to evaluate the diffusion-controlled nature of the electrochemical process, as illustrated in Figure 2(d).

Analyzing the graph, and throw linear equations: **Ipa** = −2.28 × 10^–5^ ± (1.14 × 10^–6^) + 1.57 × 10^–3^ ± (5.04 × 10^–6^) v^1/2^ (*r* ^2^ = 0.999) and **Ipc** = 2.86 × 10^–5^ ± (9.84 × 10^–7^) – 1.61 × 10^–3^ ± (6.59 × 10^–6^) v^1/2^ (*r* ^2^ = 0.999) it is possible to observe that Ipa and Ipc values rise linearly as the scan rate square root increases, proving that the Fe^3+^/Fe^2+^ redox couple is commanded by a diffusion process, with mass transport as a determinant factor of the reaction, not electron transference.^64^

Cyclic voltammetry measurements were carried out at a scan rate of 10 mV s^–1^ in 100 mmol L^–1^ sodium phosphate buffer (pH 7.4), using both a blank sample (black curve, Figure 3a) and a sample containing 1 mmol L^–1^ NADH (red curve). Experiments were conducted on the LIG sensor and a commercial SPCE for comparison. On the LIG sensor, the NADH oxidation peak appears at approximately −22 mV vs Ag/AgCl. Under identical conditions, the commercial SPCE requires a substantially higher overpotential to oxidize the NADH. As shown in Figure 3(a), the potential difference between the oxidation peaks recorded on the LIG sensor and the SPCE is approximately 330 mV vs Ag/AgCl. The peak potential observed with the fabricated sensors enables the application of amperometric detection for NADH by applying a fixed potential corresponding to the NADH oxidation peak and monitoring the resulting current aiover time. Notably, while NADH typically oxidizes at potentials above 1 V on bare electrodes,^65^ in this case, oxidation was achieved at a much lower potential, around 50 mV (end of the oxidation process on the LIG electrode). This behavior suggests that the oxygen functional groups present on the LIG surface, C=O, determined by C 1s high resolution XPS (Figure 1(d-ii)), interact with NADH as a redox mediator, promoting electrocatalytic activity toward NADH oxidation, a typical behavior, proton-dependent, previously observed for quinone groups,^56^ as can be seen in the schematic representation of Figure 3(b). The role of carbonyl oxygen groups (quinones) in enabling the electrooxidation of NADH at lower overpotentials was confirmed by fabricating LIG-based electrodes under modified laser conditions, using a lower laser fluence (303.5 mJ/cm^2^) compared to that employed in the preparation of the standard LIG electrodes used in this study (1086.8 mJ/cm^2^, as calculated in the Supporting Information). The reduced laser fluence limited the formation of carbonyl functionalities and decreased the overall content of C–O groups, due to a lower local temperature that inhibits the extensive oxidation of carbon atoms in the ambient atmosphere.^51,66,67^ This variation in chemical composition was confirmed by XPS analysis, including both survey and high-resolution C 1s spectra, as shown in Figure S2­(a–b). The electrodes fabricated under low-laser fluence conditions exhibited a reduced oxygen content (5.74%) compared to the standard LIG electrodes (10.26%), and showed the absence of C=O (carbonyl) groups, with only a small contribution from less oxidized carbon species, such as C–O groups. This limited surface oxidation led to a significantly higher electrooxidation potential for NADH, approximately ∼600 mV vs Ag/AgCl, as shown in the cyclic voltammogram of Figure S3. These findings clearly demonstrate that the presence of quinone groups is essential to promote efficient electrooxidation of NADH at low overpotentials.

**Figure 3:**
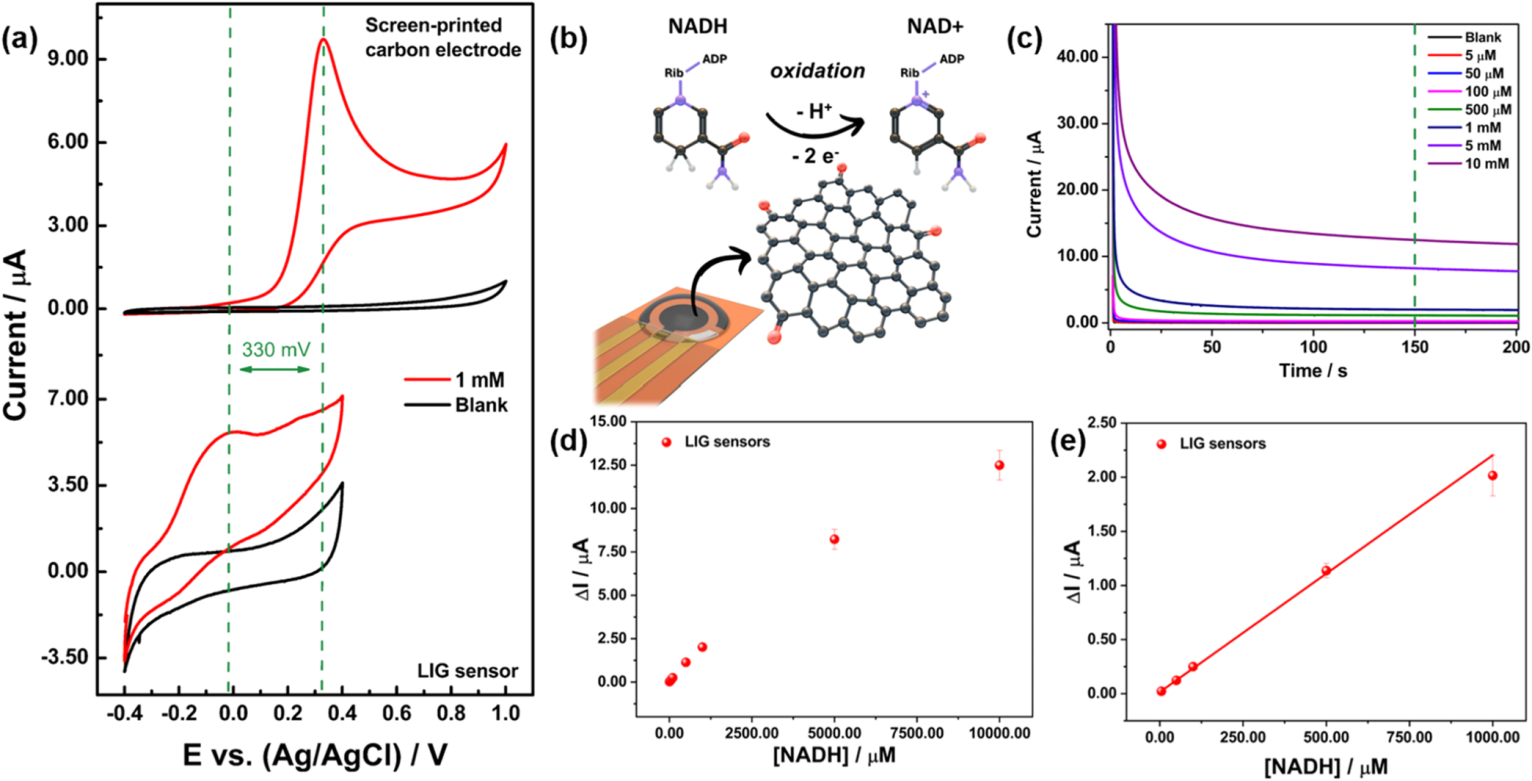
(a) Cyclic voltammetry of LIG-based sensor vs SPCE in 100 mmol L^–1^ sodium phosphate buffer (pH 7.4) comparing oxidation potential of 1 mmol L^–1^ NADH. Scan rate: 10 mV/s. (b) Schematic representation of NADH oxidation on the surface of the LIG electrode. (c) Amperometry of LIG-based sensor in 100 mmol L^–1^ sodium phosphate buffer (pH 7.4). The potential applied: 50 mV vs. printed Ag/AgCl. (d) Calibration curve of LIG-based sensor for NADH detection in 100 mmol L^–1^ sodium phosphate buffer (pH 7.4). *n* = 3. (e) The linear range of the LIG-based sensor’s calibration curve for NADH detection in 100 mmol L^–1^ sodium phosphate buffer (pH 7.4). *n* = 3.

Figure 3(c) shows the amperometric response of NADH at different concentrations, recorded at an applied potential of 50 mV vs Ag/AgCl for 200 s. On the LIG sensor, the current increases proportionally with NADH concentration, indicating effective oxidation. These results were used to construct the calibration curve for NADH detection, shown in Figure 3(d). The current values at 150 s of amperometry were used for the calibration. The linear regression equation obtained from Figure 3(e) was: LIG sensor Δ*I* (A) = 1.49 × 10^–8^ (±7.09 × 10^–9^) + 0.00219 (±7.97 × 10^–5^) [NADH] (mol L^–1^) (*r* ^2^ = 0.995). Based on these results, the sensor exhibited a linear response over the concentration range of 5 μM to 1 mM. The limit of detection (LOD), calculated as 3 × Sd _blank_/Slope for the LIG-based sensor was determined to be 2.72 μmol L^–1^, while the limit of quantification (LOQ), calculated as (10 × Sd _blank_/Slope) was 9.07 μmol L^–1^.

One of the main challenges in developing electrode materials for the electrochemical detection of NADH is their instability due to surface fouling caused by the oxidation of NADH to NAD^+^.^44^ Another critical issue is selectivity, as the electrochemical signal for NADH oxidation can be influenced by the oxidation of other species commonly present in whole blood samples, such as glucose and urea, typically found at concentrations of 5 and 7 mM, respectively.^44^ To evaluate the selectivity of the sensor, chronoamperometric measurements were performed at +50 mV vs Ag/AgCl using LIG-based electrodes for the electrochemical detection of 1 mM NADH, 5 mM glucose, 7 mM urea, and 1 mM NADH in the presence of 5 mM glucose, 7 mM urea, and both interferents simultaneously. The corresponding chronoamperograms are presented in Figure S4­(a–f). From these measurements, the variation in oxidation current (Δ*I* = *I*_a_ – *I*_blank_) for the different analyzed species is presented in Figure 4. The results demonstrate that the LIG-based sensor developed in this study exhibits high selectivity for NADH, even in the presence of potential interferents such as glucose and urea. This enhanced selectivity is attributed to the low applied potential (+50 mV vs Ag/AgCl) and the presence of carbonyl functional groups on the LIG surface, which act as quinone-like redox mediators, as previously discussed. These characteristics facilitate the selective electrooxidation of NADH while effectively suppressing the contribution of other electroactive species.

**Figure 4:**
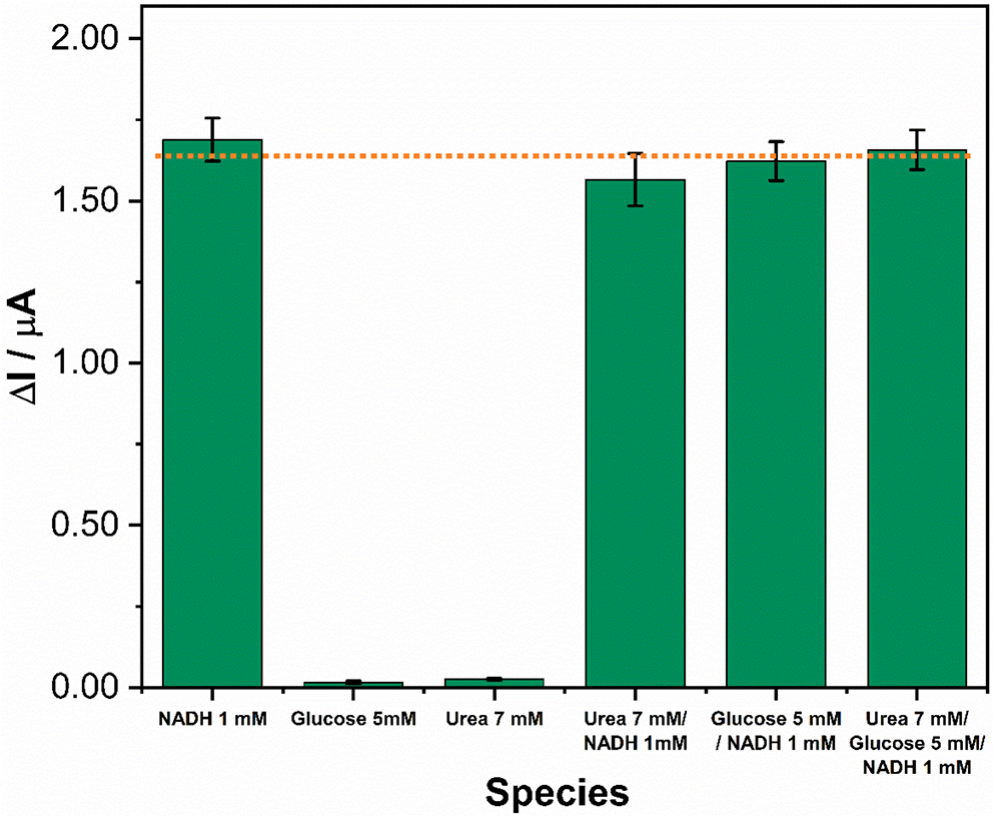
Selectivity test for the LIG-based sensor for 1 mM of NADH, 5 mM of glucose and 7 mM of urea.

Figure 5 presents the repeatability of NADH measurements and the intra- and interbatch reproducibility of the LIG-based sensor fabrication process. Repeatability was assessed by performing 10 successive chronoamperometric measurements using the same sensor for the detection of 100 μM NADH in 100 mM PBS (pH 7.4), resulting in a relative standard deviation (RSD) of 2.76%, which indicates excellent measurement consistency. Intrabatch reproducibility was evaluated by analyzing five independently fabricated electrodes (*n* = 5) produced on the same day under identical conditions, yielding an RSD of 5.78%. To assess interbatch reproducibility, three electrodes fabricated on three different days were used to detect 100 μM NADH under the same experimental conditions, resulting in an RSD of 8.22%. These results demonstrate that the LIG-based electrodes exhibit good repeatability and reproducibility, both among electrodes produced in the same batch and between different fabrication batches, highlighting their potential as a reliable platform for the development of electrochemical sensors.

**Figure 5:**
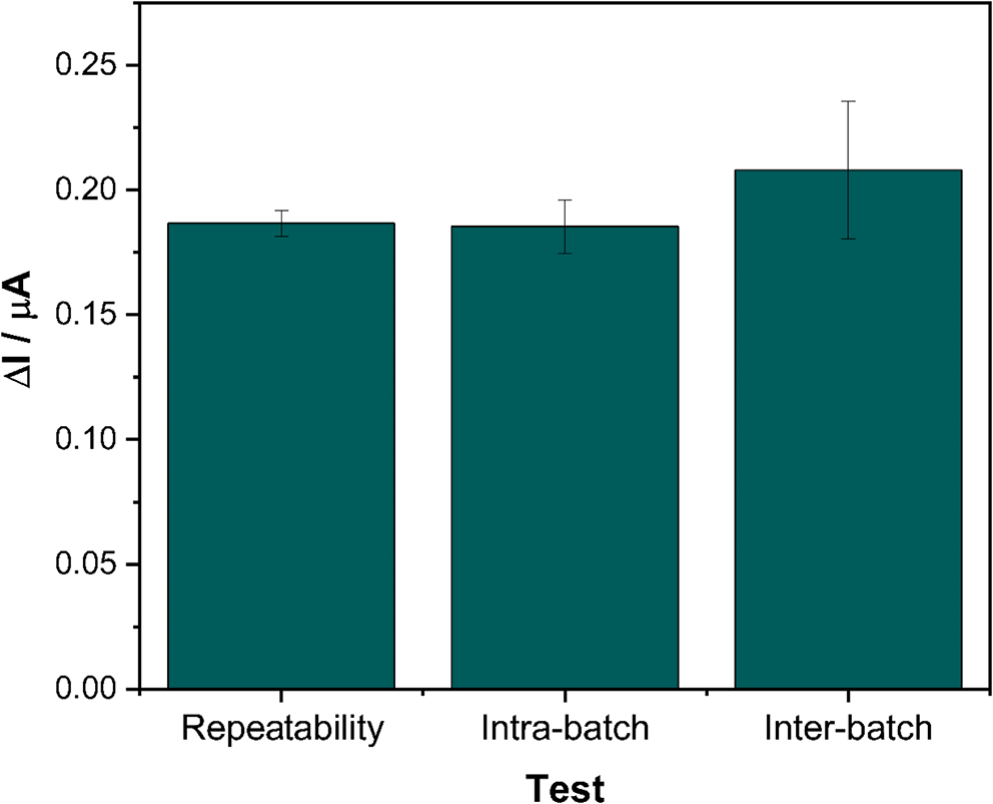
Evaluation of the repeatability and reproducibility (intra- and interbatch) of the LIG-based sensor for the detection of 100 μM NADH in 100 mM PBS (pH 7.4). Applied potential: 50 mV vs Ag/AgCl; total measurement time: 200 s.

The stability and lifetime of the electrochemical sensor are critical parameters for practical applications and were therefore thoroughly investigated. The long-term stability of the LIG-based sensors was evaluated over a one-year period by analyzing a total of 12 electrodes. At each time point, measurements were performed using three different electrodes (*n* = 3) fabricated in separate batches, following the same experimental protocol for the detection of 100 μM NADH under identical conditions. To maintain their integrity, all electrodes were stored in a desiccator under vacuum conditions (−400 mmHg) between measurements. As shown in Figure 6(a,b), a slight decrease in the current variation during the electrooxidation of 100 μM NADH was observed, with a reduction of approximately 26.24%. In addition, after one year of storage under vacuum, the relative standard deviation (RSD) for the detection of 100 μM NADH increased from 2.04 to 10.36%, indicating low loss of reproducibility over time.

**Figure 6:**
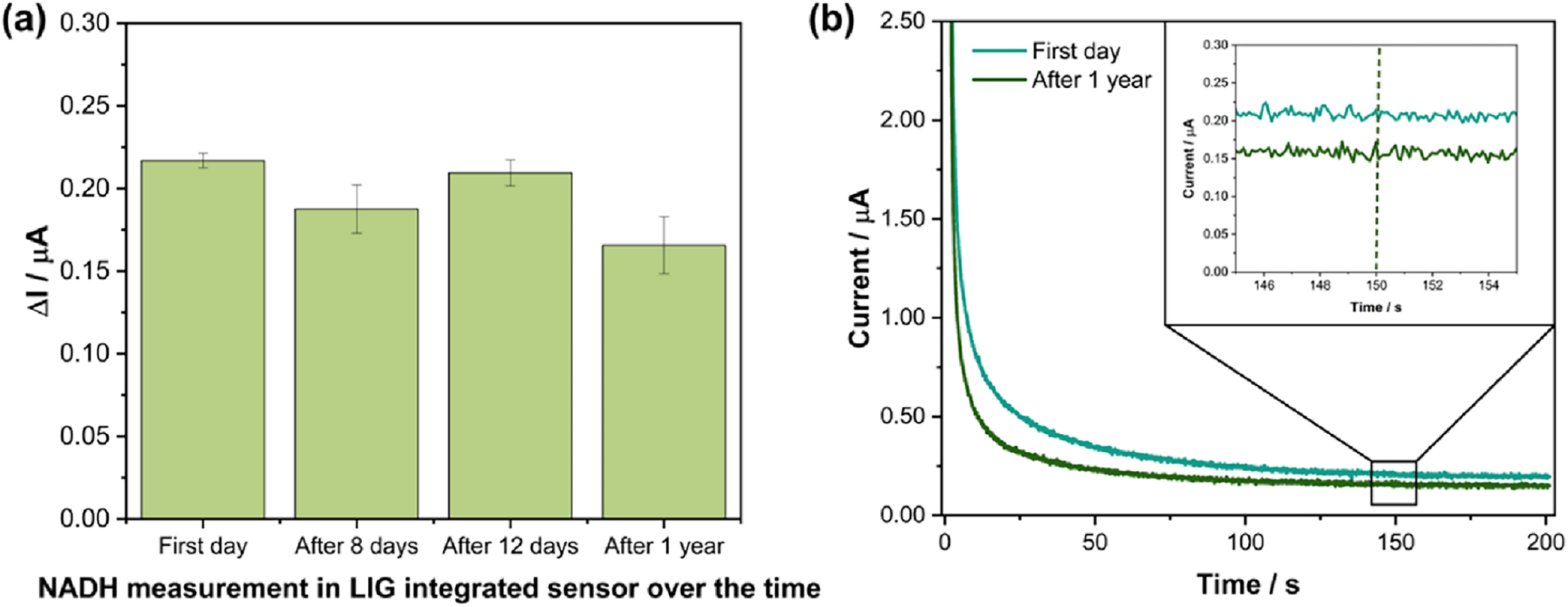
(a) Long-term stability analysis of LIG-based sensors over a one-year storage period, showing the variation in current response for 100 μM NADH. (b) Representative chronoamperometry curves for the electrooxidation of 100 μM NADH in 0.01 M PBS (pH 7.4), obtained using LIG-based sensors after different storage durations (1 day and 1 year). Applied potential: 50 mV vs Ag/AgCl; total measurement time: 200 s.

Several studies from the literature were evaluated for comparison with our results, focusing on cases where carbon electrodes were modified for NADH detection, as summarized in Table 1. Although some of these electrochemical sensors operate at relatively low potentials, they generally require additional surface modifications. In contrast, the fabricated LIG-based sensor achieved a significantly lower oxidation potential without any modification or post-treatment. In addition to the results obtained in this work, the fabrication of the LIG sensor can be considered low-cost. The unit cost for sensor fabrication is presented in Table 2.

**Table 1:** Comparison of other Carbon-Based Sensors for NADH Determination^a^

electrode	potential	linear range (μmol L^–1^)	LoD (μmol L^–1^)	refs
graphene/tungstate/GCE	–1.04 V vs Ag/AgCl	10–270	49.80	^68^
CRGO/GCE	0.45 V vs Ag/AgCl	50–650	21.85	^69^
AuNR@RGO/GCE	0.54 V vs SCE	1–31	0.22	^70^
TOA+ /PGE	0.55 V vs Ag/AgCl	1.0–15	0.077	^71^
MWCNT/3,5-DNBPh/GCE	0 V vs Ag/AgCl	100–600	22.30	^72^
PTZ/AgSNPs/SPE	0.4 V vs Ag/AgCl	1.9–89	0.76	^73^
PTZ/AgPNPs/SPE	0.4 V vs Ag/AgCl	1.9–89	0.61	^73^
PTZ/AgRNPs/SPE	0.4 V vs Ag/AgCl	1.9–89	0.52	^73^
NPG/Os(bpy)2PVI/DIA	0.35 V vs Ag/AgCl	5–100	0.80	^74^
Co-NC/Pd/CPE	0.22 V vs Ag/AgCl	5–1250	2.00	^75^
LIG	0.05 V vs Ag/AgCl	5–1000	2.72	this work

a CRGO- chemically reduced graphene oxide, AuNR-Gold nanorods, SCE- saturated calomel electrode, RGO-reduced graphene oxide, MWCNT -Multiwall carbon nanotube, TOA+- tetraoctylammonium ions, PGE-pencyl graphite electrode, 3,5 DNBPh −4-phenylbutyl-3,5-dinitrobenzoate, SPE-screen-printing electrode, GCE-glassy carbon electrode, AgPNPS-Silver nanoprism, AgRNPs-Silver nanorods, AgSNPs-Silver nanospheres. GCE-glassy carbon electrode, SPE-Screen-printing electrode, NPG-Gold nanoporous Os­(bpy)­2­(PVI)-osmium-based polymer, DIA-Diaphorase, Co-NC/Pd-Cobalt-N-doped carbon and palladium, CPE-carbon paste electrode.

**Table 2:** Estimated Material Costs for LIG Sensor Fabrication^a^

material	value in USD ($)	value per sensor ($)
polyimide	249.96	0.013
Kapton tape	4.45	0.002
conductive silver paint	78.00	0.07
bleach	2.18	0.00002
nail polish	1.31	0.01
total value per sensor		0.10

a *Laser Engraver (3W, 450 nm) cost: $253.41.

The cost per sensor for the polyimide was calculated based on the area used to fabricate each sensor, while the costs of the other materials were estimated considering their use in the fabrication of approximately 200 sensors. The total cost for the fabrication of each electrochemical cell was U$ 0.10.

## 4 Conclusions

In this work, we successfully demonstrated the fabrication of a low-cost, in-house laser-induced graphene (LIG) sensor for the amperometric detection of NADH. The LIG/polyimide (PI) composite, produced via 450 nm laser irradiation, exhibited excellent electrical conductivity, as confirmed by Raman spectroscopy, XPS, and sheet resistance measurements. The presence of C=O functional groups on the LIG surface likely enhanced the electrocatalytic oxidation of NADH, enabling detection at a remarkably low applied potential of 50 mV vs Ag/AgCl. The sensor showed high sensitivity, with a limit of detection of 2.72 μmol L^–1^, a limit of quantification of 9.07 μmol L^–1^, and a wide linear range up to 1 mmol L^–1^. The repeatability of the electrochemical oxidation of NADH resulted in a relative standard deviation (RSD) of 2.76%, while the reproducibility, evaluated as intra- and interbatch variability, yielded RSD values of 5.78 and 8.22%, respectively. In addition to its strong analytical performance, the low fabrication cost of approximately U$ 0.10 per device highlights its potential for scalable, sustainable, and eco-friendly electrochemical sensing. These results position the LIG-based platform as a promising candidate for detecting NADH and, by extension, for broader applications in biomedical diagnostics and environmental monitoring.

## Supplementary Material



## Data Availability

The authors declare that the data supporting the findings of this study are available within the paper. Should any raw data files be needed in another format, they are available from the corresponding author upon request.

## Notes

Equation for laser fluence calculation; stability of Ag/AgCl pseudoreference electrodes; XPS spectra (survey and C 1s) of LIG (82.5 mW, 17 mm/s, two passes, air); cyclic voltammetry of 5 mM NADH (PBS, pH 7.4) on LIG (82.5 mW, 17 mm/s, two passes, air) sensor; chronoamperograms for interferent studies (PDF)
