# Supplementary material for: Cost-Effective Fabrication of Laser-Induced Graphene Electrochemical Cell for NADH Detection
Source: ACS Omega. 2025 Oct 9;10(41):48100–10. doi: 10.1021/acsomega.5c04282 (PMC12547605; doi:10.1021/acsomega.5c04282)
Supplement: Supplementary file 1 [file ao5c04282_si_001.pdf]

Supplementary Electronic Materials for

Cost-Effective Fabrication of Laser-Induced Graphene Electrochemical Cell for NADH Detection

Ketley Caroline Rocha Pereira [1,2], Elsa Maria Materón [1,2], Matheus Santos Dias[1,2], Tatiana Parra Vello[2], Deissy Feria Garnica[2], Gustavo Miguel Sousa[1,2], Camila Marchetti Maroneze[1,2] and Cecilia de Carvalho Castro Silva[1,2,*]

[1] *School of Engineering, Mackenzie Presbyterian University, São Paulo 01302-907, São Paulo, Brazil*

[2] *MackGraphe – Mackenzie Institute for Research in Graphene and Nanotechnologies, Mackenzie Presbyterian Institute, São Paulo 01302-907, São Paulo, Brazil*

* Corresponding author: Tel: +55 -11 -27667380.

E-mail: cecilia.silva@mackenzie.br (C.C.C.S)

This document file includes:

Supplementary Text

Figs. S1 to S4

Equation employed to calculate the laser fluence:

$$\textit{Equation 1}\ .\ \mathbf{F_{pulse}} = \frac{P \times \tau}{A}$$

F_{pulse} = pulse fluence (J/cm^2)

P = laser power (W)

τ= pulse duration (s) = 4.16×10^{-4} s

A = beam area (cm^2) = 1.13×10^{-4} cm^2

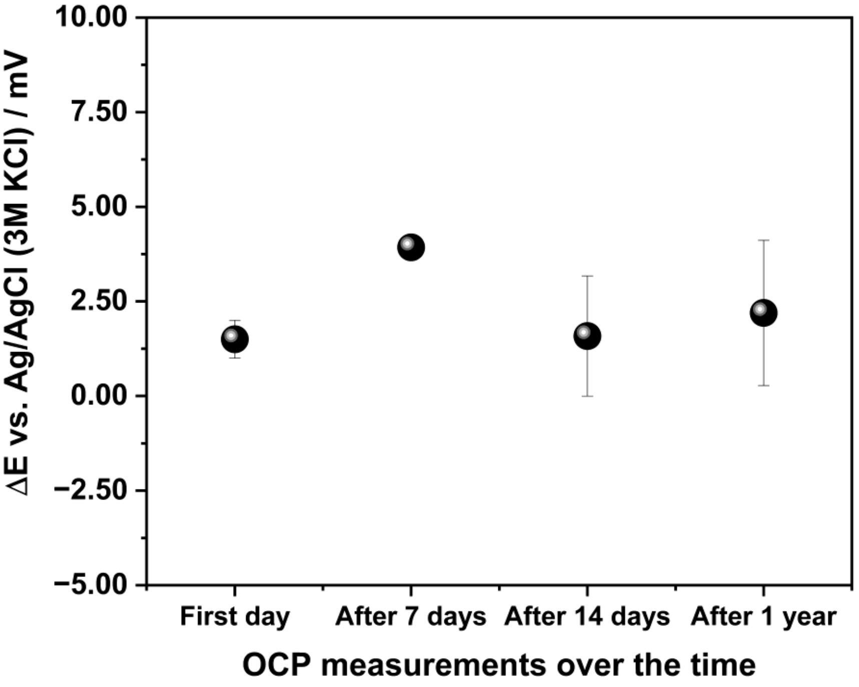

Figure S1. Open-circuit potential (OCP) variation of the fabricated Ag/AgCl electrodes measured against an external Ag/AgCl (3 M KCl) reference electrode over storage periods of 7 days, 14 days, and 1 year (n=3); total measurement time: 800 seconds.

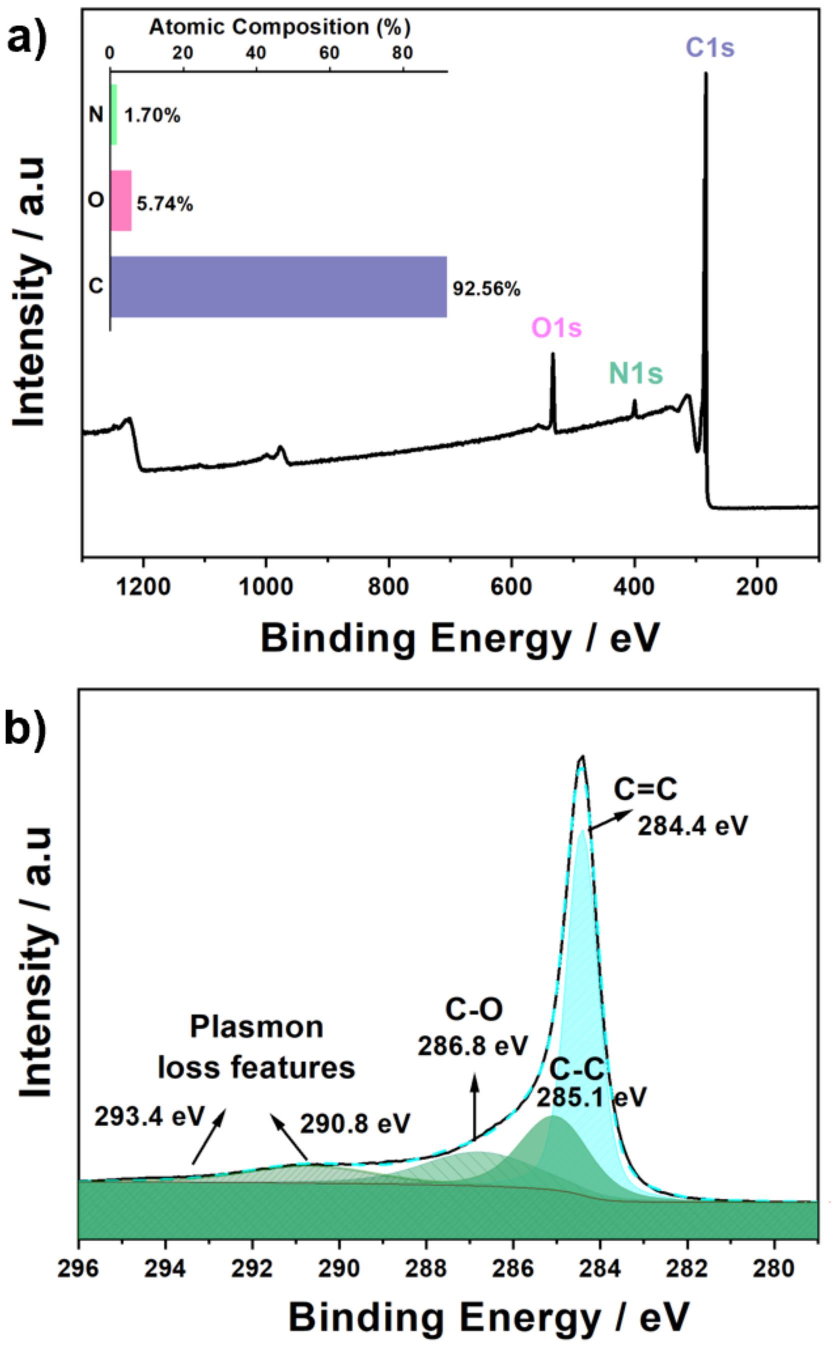

Figure S2. Representative XPS analysis of LIG sample prepared using laser power of 82.5 mW, engraving speed of 17 mm/s, and two processing passes, all performed in ambient air. Survey spectrum with elemental composition analysis (a) and high-resolution spectrum for C 1s (b).

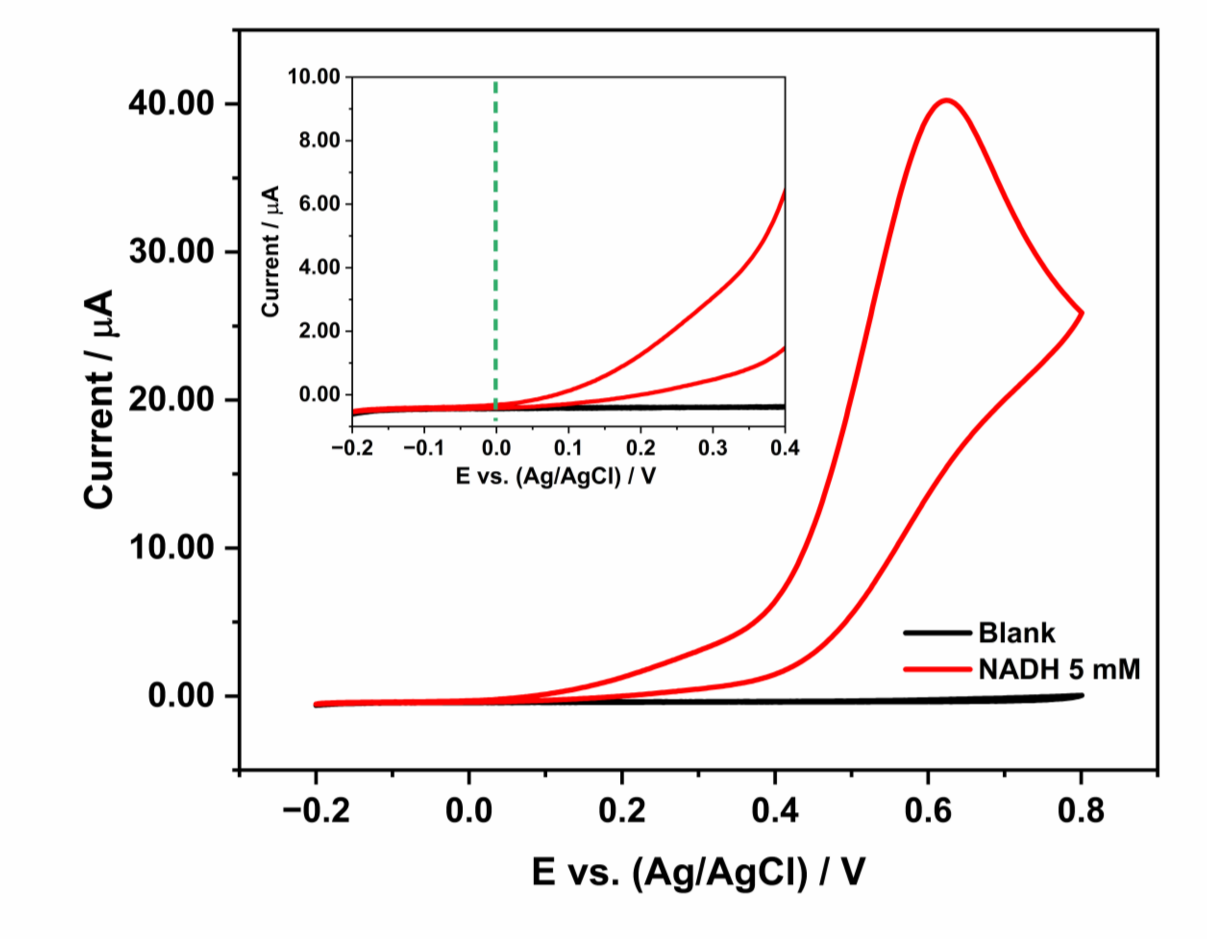

Figure S3. Cyclic voltammetry of NADH 5 mM in 0.01 M phosphate-buffered saline (pH 7.4) was performed on the LIG fabricated sensor using laser power parameters of 82.5 mW, engraving speed of 17 mm/s, and two processing passes, performed under ambient air. Insert: Magnification of the respective cyclic voltammogram in the region around 0 mV. Scan rate: 10 mV/s.

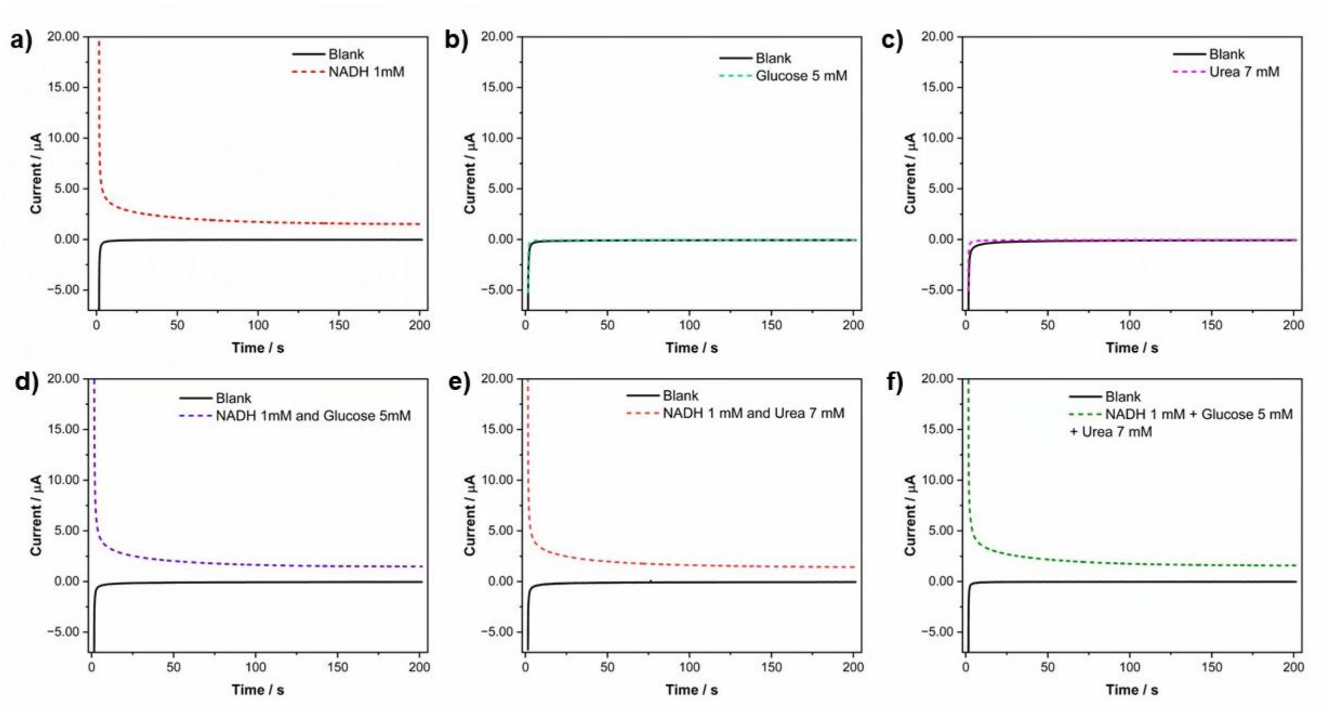

Figure S4. Representative chronoamperometry curves of the LIG-based sensor in the presence of 1 mM NADH and potential interferents (5 mM glucose and 7 mM urea) in 0.01 M phosphate-buffered saline (PBS), pH 7.4. Applied potential: 50 mV; total measurement time: 200 seconds.
